# Evaluation of the effect of photoplethysmograms on workers’ exposure to methyl bromide using second derivative

**DOI:** 10.3389/fpubh.2023.1224143

**Published:** 2023-09-25

**Authors:** Jungmi Choi, Wonseok Cha, Min-Goo Park

**Affiliations:** ^1^Human Anti-Aging Standards Research Institute, Uiryeong-gun, Gyeongsangnam-do, Republic of Korea; ^2^Department of Bioenvironmental Chemistry, Jeonbuk National University, Jeonju, Republic of Korea

**Keywords:** fumigant, methyl bromide, fumigation worker safety, second derivative wave, photoplethysmogram, urinary bromide concentration

## Abstract

Methyl bromide (MB) is worldwide the only effective fumigant heavily used for quarantine pre-shipment treatment and has a critical use exemption for soil fumigations due to its excellent permeability and insecticidal effect. However, MB should be replaced as it is an an ozone-depleting substance and also highly toxic to humans. Recently, MB has been shown to be hazardous even for asymptomatic workers, affecting their central and autonomic nervous systems. However, the effects of MB exposure on vascular health have not been explored. This study aimed to determine whether MB affects the arterial system of asymptomatic workers. We measured the second derivative of the photoplethysmogram (SDPTG) indices, which are indicators of vascular load and aging, and urinary bromide ion (Br^−^) concentrations in 44 fumigators (study group) and 20 inspectors (control group) before and after fumigation. In fumigators, the mean values of post-work SDPTG indices (b/a, c/a, d/a, e/a, and SDPTG aging index) and Br^−^ levels were significantly changed compared to their pre-work values (*p* < 0.05), indicating a negative effect on their cardiovascular health. In contrast, SDPTG indices and Br^−^ levels in inspectors did not show any differences before and after work. All SDPTG indices except c/a showed significant correlations with Br^−^ levels in all individuals (*p* < 0.05). In conclusion, the Br^−^ levels and SDPTG indices of fumigators varied after MB work, and they experienced negative effects on their health despite being asymptomatic.

## 1. Introduction

Methyl bromide (MB) is a fumigant used worldwide for controlling pests in soils and agricultural products, including grains, fruits, and wood, owing to its efficacy against pests and its permeability to commodities (1–3). Although MB should be phased out due to it has been identified as an ozone-depleting substance under the Montreal Protocol, MB currently is still a world-wild recognized effective fumigant and heavily used for quarantine pre-shipment treatment and a critical use exemption for soil fumigations (4).

MB is also highly toxic to humans. It is mostly absorbed into the body by inhalation, which leads to peripheral neurological disturbances (5), central neurological effects (6–9), or swallowing disorders (10). Recent studies on asymptotic workers exposed to MB have reported negative effects on the central and autonomic nervous systems (11, 12). Moreover, acute coronary syndrome occurred in a patient exposed to MB from an old fire extinguisher, leading to cardiogenic shock (13). Therefore, concerns of work safety and health issues are still a challenge to those who conduct MB fumigation and inspect MB-fumigated shipping container and cargoes (14).

Bromide ion (Br^−^) concentrations have been utilized as biomarkers in workers exposed to MB, with Br^−^ detected in both blood and urine samples (15–17). Nevertheless, individuals with high MB concentrations can remain asymptomatic (16, 18). One possible explanation is the variation in symptom development among individuals (18). Therefore, we utilized Electroencephalogram and heart rate variability as sensitive markers of nervous function, confirming damage to the central and autonomic nervous systems in exposed individuals (11, 12). However, the impact of MB fumigation on cardiovascular health, including arterial function, remains unexplored. Biomarkers that can effectively assess vascular health have not yet been identified in workers exposed to MB.

Vascular load and aging are typically evaluated using the second derivative of the photoplethysmogram (SDPTG), which can be a valuable parameter in determining the vascular effects of conditions such as hypertension, atherosclerosis, and aging (19–21). The pulse wave in the photoplethysmogram (PTG) method measures changes in the amount of light absorbed by hemoglobin and generally reflects a change in blood volume (22–25). Pulse wave changes provide more information on blood pressure than simple systolic-diastolic blood pressure measurements (20) and have, therefore, been used to evaluate the elasticity of arterial vessels. However, detailed shape information such as the inflection points in the PTG is difficult to grasp visually. The SDPTG, developed by utilizing second derivative waves of the PTG to enhance recognition accuracy, has since been widely adopted as an objective and dependable measurement technique (20, 26–28). The SDPTG-derived indices exhibit a linear correlation with age and, thus, serve as a valuable tool for evaluating the decline in vascular function (20, 29, 30). We developed in a previous study (31) a linear regression model to analyze various SDPTG indices (b/a, c/a, d/a, e/a, and the SDPTG aging index [SDPTG-AI] based on the age of Korean participants) and used identical instruments and protocols in the current study. The findings of our previous study indicate that these SDPTG indices could be useful and sensitive tools for assessing arterial deterioration.

This study aimed to evaluate the potential impact of occupational exposure to MB on the health of workers. SDPTG levels were measured both before and after fumigation, and traditional biomarker analysis of Br^−^ in urine was conducted to assess whether workers had been exposed to MB and to quantify the extent of exposure.

## 2. Materials and methods

### 2.1. Participants

This study included 44 participants who were employed by pest control companies that utilized MB and were registered with the Animal and Plant Quarantine Agency (APQA) in Korea (32). The control group consisted of 20 public officials from the Youngnam Regional Office of the APQA in Busan, Korea, who were responsible for overseeing the MB work.

Between February and August 2019, SDPTG indices were assessed in one or two individuals from each group on the day of MB fumigation. All participants underwent screening for SDPTG by registered clinical nurses, and bromine ion concentrations in urine were monitored both before and after fumigation. The fumigators in this study were responsible for sealing the containers, measuring the MB concentrations, administering the MB, and ventilating the area following MB application. In contrast, the quarantine inspectors oversaw the fumigation process but were less likely to experience direct MB exposure due to their different work tasks. All workers wore gas masks attached to a respirator canister for air purification during MB fumigation work, whereas not all inspectors wore masks, based on the relevant phytosanitary regulation (33). For further information regarding the work and SDPTG measurement procedures of the participants please see Supplementary Table S1.

This observational study was conducted as a component of the APQA’s plant quarantine technology development program and was authorized by the Institutional Review Board of Dong-A University (IRB number: 2-1040709-AB-N-01-201806-BR-004-04). All participants provided written informed consent for study participation and analysis of all collected data.

### 2.2. Analysis of bromide ions in urine

Urine samples were collected after the participants were instructed about the proper collection method to avoid contamination. After discarding the first portion of urine, over 10 mL of intermediate urine was obtained using a designated urine cup (Qorpak PLC-03701 Natural Polypropylene Jar with 58–400 White Polypropylene Unlined Cap 120 mL) and kept at 4°C until being transported for testing. Each transferred sample (5 mL) was placed into special conical tubes (CELLTREAT 229412 Centrifuge Tube, 15 mL, polypropylene) and stored in a freezer at −80°C for analysis of the Br^−^ concentration. The Br^−^ concentration in urine was measured using a high-performance liquid chromatography-inductively coupled plasma mass spectrometer (HPLC/ICP-MS) system, consisting of Agilent Technologies 1,260 Series HPLC and Agilent Technologies 7,700 Series ICP-MS, both made by Agilent Technologies (CA, United States).

### 2.3. SDPTG measurements

The study participants were instructed to sit in a relaxed position with their eyes open, and their pulse waves were monitored for 5 min using a photoplethysmometer (34). A PTG sensor (model: ubpulse T1 [Pulse Analyzer, KFDA Certification No. 11–1,296], LAXTHA Inc., South Korea) was placed on the left index finger of each participant (34).

The hand with the sensor was placed on a table at chest height, with nail polish or other visual obstructions removed from the finger (35). To avoid interference, the participants were asked to remove clothing or items that put pressure on their arms or fingers, such as sleeves, bands, or rubber bands. The participants were also advised not to breathe deeply or abdominally during the measurements, to prevent respiratory sinus arrhythmias (35).

**Figure 1:**
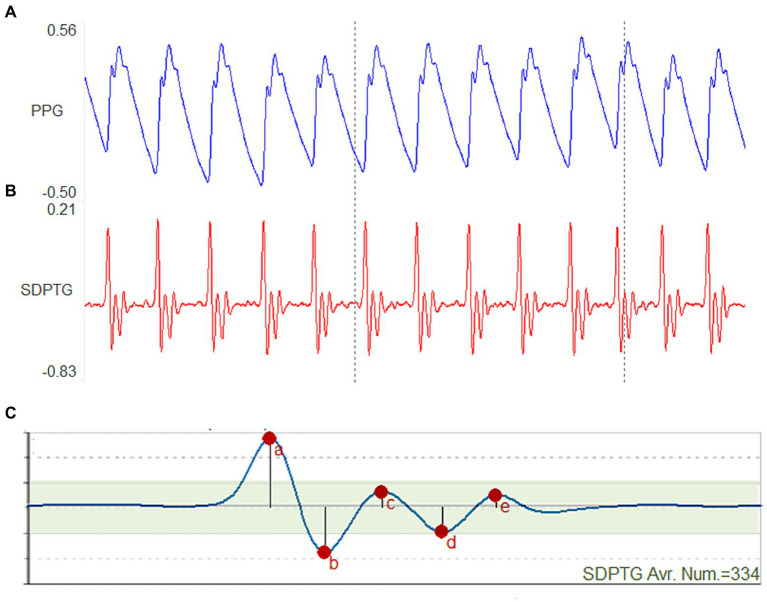
Photoplethysmogram (PTG) and second derivative photoplethysmogram (SDPTG) signals measured in one participant. **(A)** An 8-s PTG wave. **(B)** An 8-s SDPTG wave. **(C)** The average wave of 334 SDPTG waves over 5 min.

The device was used to perform PTG and SDPTG measurements simultaneously, with the SDPTG waveform displayed after the PTG derivative, as shown in Figure 1. The operator monitored the signal shape to minimize data contamination and ensured that it was not disrupted by finger or sensor movements.

### 2.4. Biomarkers and calculations of SDPTG

The SDPTG waveform was calculated as the second derivative of the PTG wave. The SDPTG waveform offers a more comprehensive depiction of the PTG characteristics, encompassing crucial details such as inflection points and multiple positive and negative peaks, as illustrated in Figure 1. Each SDPTG complex consists of five waves, namely “a,” “b,” “c,” “d,” and “e,” which are defined as follows:

1. The “a” wave is the first large positive peak that appears in the SDPTG signal.
2. The “b” wave is the negative peak that immediately follows the “a” wave.
3. The “c” wave is the positive peak that immediately follows the “b” wave.
4. The “d” wave is the negative peak that immediately follows the “c” wave.
5. The “e” wave is the positive diastolic peak that appears immediately after the “d” wave.

Figure 1 illustrates how the a, b, c, d, and e waves correspond to the amplitude values of each peak in the SDPTG. To perform SDPTG waveform analysis, the relative amplitudes of the b, c, d, and e waves with respect to that of the a wave were calculated. The resulting ratios b/a, c/a, d/a, and e/a were used as SDPTG indicators in this study. To reflect the functional aging level of the arteries, SDPTG-AI was determined using the second derivative of the PTG. It was calculated as a combination of b/a, c/a, d/a, and e/a ratios, weighted by +1 or − 1 depending on their proportional or inverse relationship with the functional aging of blood vessels. Specifically, the ratio b/a tends to increase with age, whereas the ratios c/a, d/a, and e/a decrease with age. By combining these ratios, SDPTG-AI is considered to be a reliable predictor of arteriosclerosis, a common condition associated with substantial functional aging of blood vessels (20, 36, 37).

To accurately determine the SDPTG indices, it is important to use pulse data free of ectopic beats, arrhythmic events, noise, and missing data (38). Therefore, only clean pulse data were used in this study.

### 2.5. Statistical analysis

SDPTG and urinary Br^−^ concentrations in each group are presented as the mean and standard deviation (SD). To compare the baseline characteristics of the participants, independent *t*-tests were conducted for continuous variables, and chi-square tests were performed for categorical variables. Independent *t*-tests were used to compare SDPTG and Br^−^ levels between the study and control groups before and after fumigation. A paired *t*-test was performed to determine differences in SDPTG and Br^−^ levels before and after fumigation within each group. The effect of MB fumigation on SDPTG and Br^−^ levels between the groups was evaluated using analysis of covariance, controlling for pre-work SDPTG and Br^−^ levels. The relationship between urinary Br^−^ concentration and SDPTG indices for all participants was analyzed using Pearson’s correlation while controlling for age and sex through partial correlation. Data analysis was conducted using SPSS version 23, and a significance level of *p* < 0.05 was used.

## 3. Results

### 3.1. Demographic information

An overview of the demographic information and starting values of study participants is provided in Table 1. The participants in the fumigation group were mostly male, older, and had a higher rate of smoking than those in the inspection group. The use of gas masks was more prevalent in the inspection group on the test days. However, other factors, such as work duration and alcohol consumption were not significantly different between the two groups.

**Table 1:** Demographic information.

Demographic variable	Fumigator	Inspector	*p*-value
Sex: Male	44 (100%)	13 (65.0%)	<0.001
Age (years)	42.54 ± 10.19	37.15 ± 10.81	0.059
Alcohol: Yes	42 (95.5%)	18 (90.0%)	0.403
Smoking: Yes	19 (43.2%)	5 (25.0%)	<0.001
Duration of work (years)	10.15 ± 9.62	6.65 ± 8.06	0.161
Gas mask^a^ use on test day: Yes	44 (100%)	13 (65.0%)	<0.001

a Gas masks used by participants during MB fumigation work were attached to a canister for air purification. These masks were full-face masks designed to purify various toxic gases, including organic vapor (OV), and were manufactured by 3 M in Seoul, South Korea.

The data are summarized as the mean ± SD for continuous variables (age and duration of work) and the frequency and proportion for categorical variables (sex, smoking, alcohol, and gas mask use on the test day). *p*-values were derived from the independent *t*-test for continuous variables and Fisher’s exact test for categorical variables. MB, methyl bromide; SD, standard deviation.

### 3.2. Comparison of SDPTG indices and urinary Br^−^ concentration before and after MB work by group

**Figure 2:**
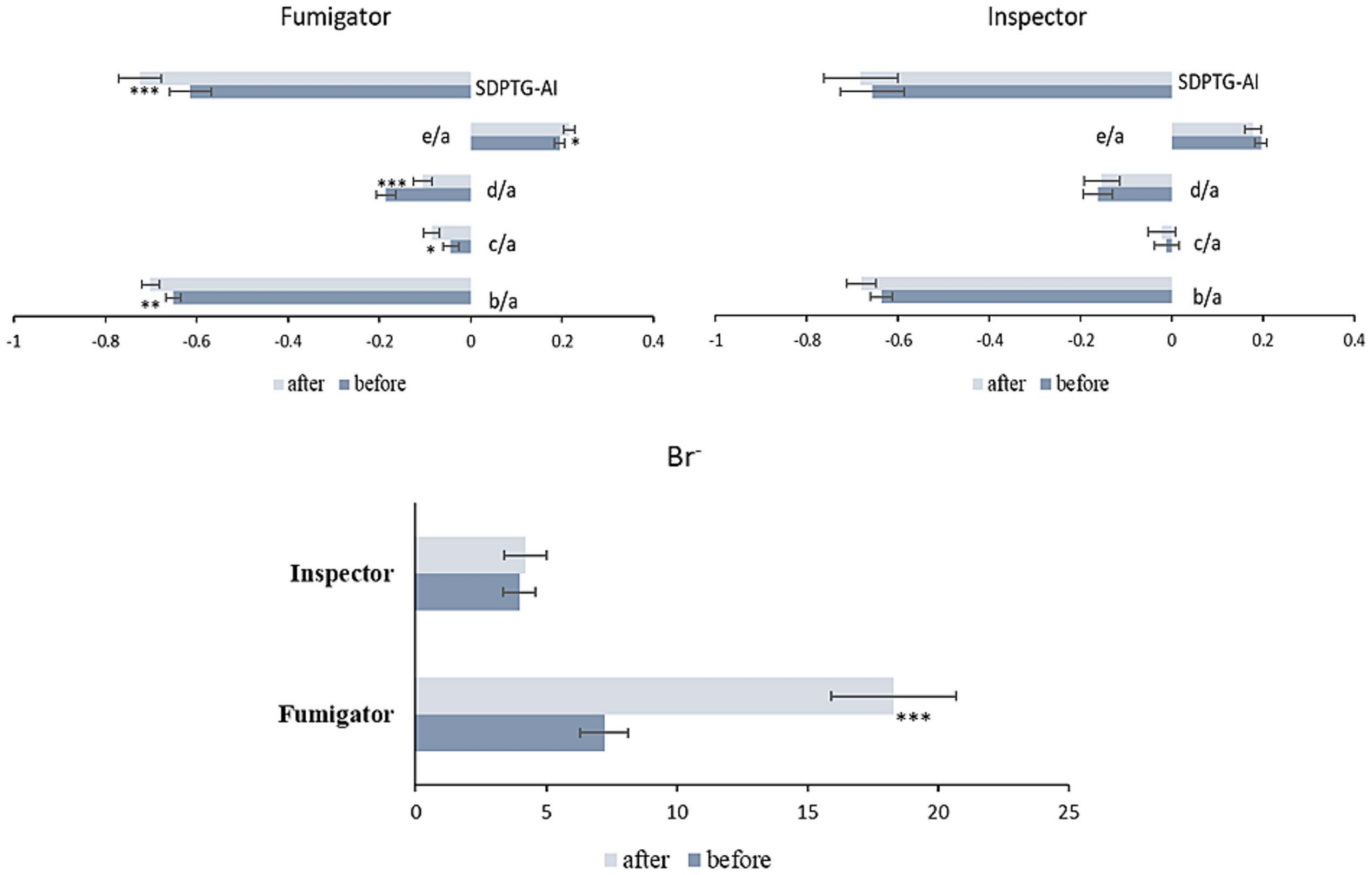
Changes in SDPTG indices and Br^−^ levels of fumigators and inspectors before and after fumigation work. ^*^p < 0.05, ^**^p < 0.01, and ^***^p < 0.001. Br^−^, bromide ion; SDPTG-AI, second derivative of the photoplethysmogram - aging index.

The changes in SDPTG indices and urinary Br^−^ concentrations over time by group are summarized in Table 2; Figure 2. The results showed that the SDPTG indices b/a, c/a, d/a, e/a, and SDPTG-AI for the fumigators changed significantly after work compared to those before work. The b/a, c/a, and SDPTG-AI values decreased, whereas the d/a and e/a values increased. However, the inspectors did not show any significant level differences between before and after work. The mean urinary Br^−^ concentrations before work in fumigators and inspectors were 7.39 and 3.98 μg/mg creatinine, respectively, and these were significantly different (*p* = 0.006). After work, the mean urinary Br^−^ concentrations increased to 18.31 and 4.21 μg/mg creatinine, respectively, and this difference between the two groups was also significant (*p* < 0.001). The mean urinary Br^−^ concentration in the fumigators significantly increased after work compared to that before work (*p* < 0.001); however, this was not the case for the inspectors.

**Table 2:** SDPTG indices and urinary Br^−^ concentrations of fumigators and inspectors before and after MB work.

Index	Fumigator (*n* = 44)		*p*-value	Inspector (*n* = 20)		*p*-value
	Before	After		Before	After	
Br^−^ (μg/mg CRE)	7.39 ± 6.47	18.31 ± 16.00	<0.001	3.98 ± 2.83	4.21 ± 3.64	0.770
b/a	−0.65 ± 0.10	−0.70 ± 0.13	0.003	−0.64 ± 0.11	−0.68 ± 0.15	0.131
c/a	−0.04 ± 0.12	−0.09 ± 0.12	0.019	−0.01 ± 0.12	−0.02 ± 0.14	0.775
d/a	−0.19 ± 0.14	−0.11 ± 0.14	<0.001	−0.16 ± 0.15	−0.15 ± 0.18	0.821
e/a	0.19 ± 0.07	0.21 ± 0.08	0.010	0.20 ± 0.06	0.18 ± 0.08	0.296
SDPTG-AI	−0.61 ± 0.31	−0.72 ± 0.31	<0.001	−0.66 ± 0.32	−0.68 ± 0.37	0.686

Urinary Br^−^ concentration and SDPTG indices are expressed as the mean ± SD. *p*-values were calculated using the paired *t*-test. Br^−^, bromide ion; CRE, creatinine; MB, methyl bromide; SD, standard deviation; SDPTG-AI, second derivative of photoplethysmogram - aging index.

The trend between the two groups after work compared to before work showed significant differences for d/a, e/a, and urinary Br^−^ when the pre-work SDPTG values and Br^−^ ions were controlled for using analysis of covariance: d/a (*F* (1,61) = 4.257, *p* = 0.043), e/a (*F* (1,61) = 6.016, *p* = 0.017), Br^−^ (*F* (1,61) = 9.232, *p* = 0.003). However, the b/a, c/a, and SDPTG-AI trends were not significantly different between the groups.

### 3.3. Correlation between bromide ion levels and SDPTG indices in all individuals

**Figure 3:**
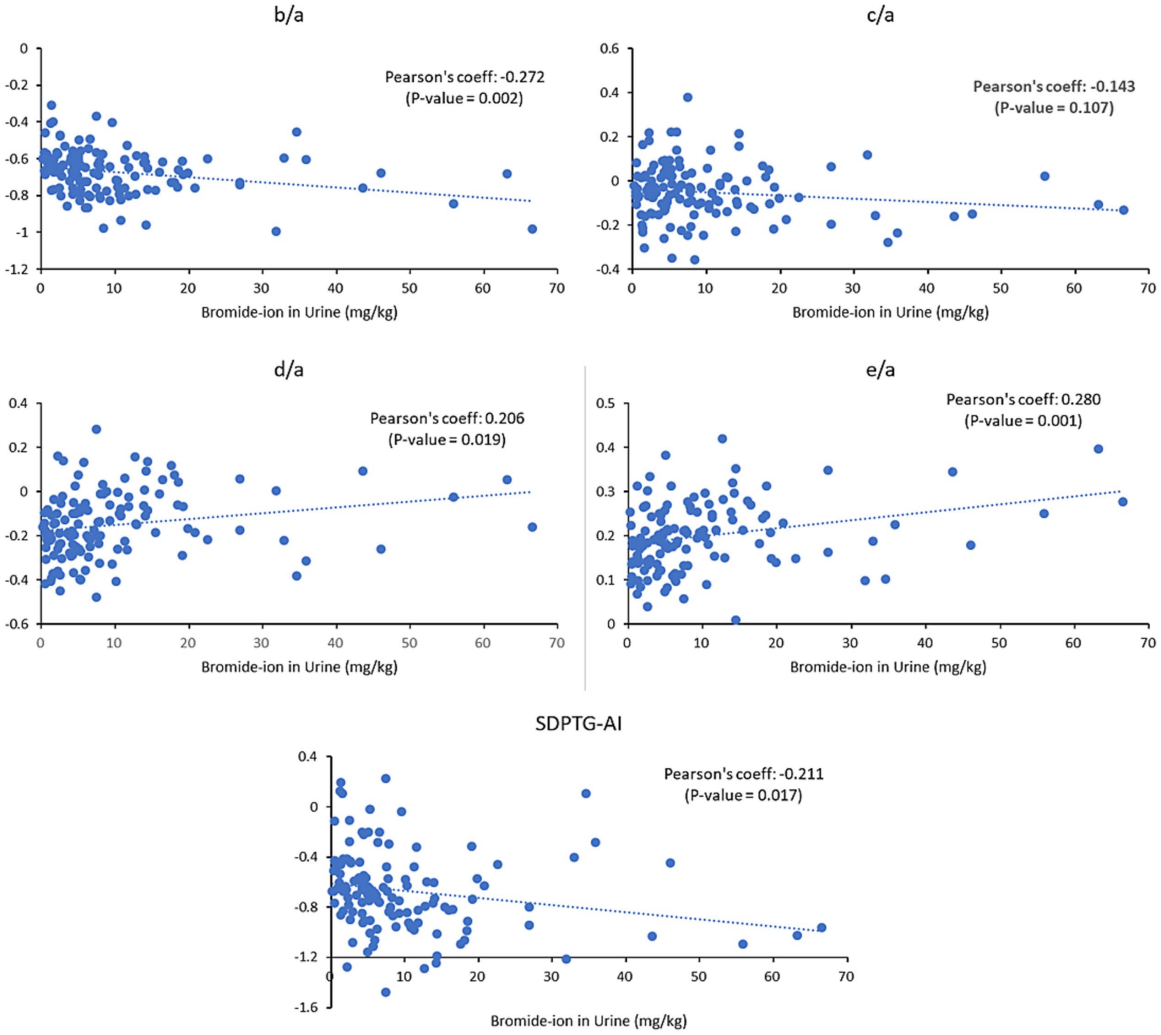
Correlation between urinary Br^−^ levels and SDPTG indices in all participants before (*n* = 64) and after (*n* = 64) work. There appears to be a correlation between Br^−^ concentrations and all SDPTG indices except c/a. Br^−^, bromide ion; SDPTG-AI, second derivative of the photoplethysmogram - aging index.

The relationships between the levels of urinary Br^−^ and SDPTG indices were examined for all 64 participants before and after work, without differentiation between the two groups. The results showed a linear correlation between urinary Br^−^ and all SDPTG values (b/a, d/a, e/a, and SDPTG-AI), except c/a, with a *p*-value less than 0.05. The results indicate that b/a and SDPTG-AI decreased as the levels of Br^−^ increased, whereas d/a and e/a increased as the levels of Br^−^ increased. The partial correlation analysis controlling for age and sex confirmed these findings, with the correlation magnitude increasing for all indices, except b/a, compared to that of the uncontrolled analysis. The complete correlation analysis is presented in Table 3 Figure 3.

**Table 3:** Correlations of urinary Br^−^ levels and SDPTG indices among all participants before and after work with and without controlling for age and sex.

Control variables		b/a	c/a	d/a	e/a	SDPTG-AI
None^a^	Correlation	−0.272	−0.143	0.206	0.280	−0.211
	*p*-value	0.002	0.107	0.019	0.001	0.017
	d.f.	126	126	126	126	126
Age and sex	Correlation	−0.265	−0.169	0.250	0.295	−0.256
	*p*-value	0.003	0.058	0.005	0.001	0.004
	d.f.	124	124	124	124	124

a Cells contain zero-order (Pearson) correlations. The numbers of participants were 64 before and after work.

Br^−^, bromide ion; SDPTG-AI, second derivative of photoplethysmogram - aging index.

## 4. Discussion

SDPTG has been employed to quantitatively evaluate vascular health in relation to diseases such as hypertension, atherosclerosis, and aging in many studies (21, 37, 39–41). Specifically, a clear regression model of various SDPTG indices has been recently presented with age in healthy Korean adults, and thus reference ranges have been supplied depending on the age groups (31): SDPTG indices exhibited an age-related increase in b/a and SDPTG-AI while showing a decrease in c/a, d/a, and e/a. In the present study, SDPTG indices were used to measure the effects of MB on humans. The mean values of the SDPTG indices in the fumigator group after MB work were significantly different from those before work: b/a, c/a, and SDPTG-AI decreased after work, whereas d/a and e/a significantly increased (Table 2 Figure 2). Except for c/a, values for SDPTG in the fumigator group after work changed in reverse with aging even if the mean concentration of urinary Br^−^ in the fumigator increased after work. The SDPTG indices of the inspectors in the control group did not change significantly (Table 2 Figure 2).

Only the parameter c/a did not follow the same pattern as that of the other indices. It should be excluded due to the nonsignificant correlation between SDPTG indices and Br^−^. All SDPTG indices except c/a showed correlations with urinary Br^−^ levels, in all individuals before and after work (Table 3; Figure 3).

Pulse waves, the raw data of SDPTG, reflect the pressure in the ascending aorta. This pressure can be divided into two components (20). The initial systolic component is attributed to the left ventricular ejection, whereas the subsequent component is amplified by peripheral reflection waves (20, 42). Among the SDPTG indices, the b/a ratio reflects the early systolic component, which is lower when the vascular wall has a low vascular load. The d/a ratio reflects the second component and is higher when the peripheral arterial wall has a low vascular load (20). The c/a ratio, calculated using the c wave, which is a connected wave between the b and d waves, is higher in the young arterial wall or with a low vascular load (28). The e/a ratio reflects the initial rise in the diastolic wave, which is higher in young and flexible arterial walls or under low vascular loads (20). The SDPTG-AI, calculated as b/a-c/a-d/a-e/a, indicates that the functional aging level of the artery is lower in young and flexible artery walls or under low vascular loads (31). A low vascular load can be caused by high flexibility or poor left ventricular ejection. Considering that an old man exposed to MB had a cardiogenic shock with a left ventricular ejection fraction of 30% (13), it can be presumed that MB exposure lowered the b/a and SDPTG-AI of fumigators while raising their d/a and e/a values due to decreased left ventricular ejection. Therefore, we can infer that the inverse alteration in SDPTG values within the fumigator group as they age, despite an increase in the mean concentration of urinary Br- after work, may be attributed to a reduction in left ventricular ejection due to MB exposure.

In this study, MB was found to negatively affect the cardiovascular health of fumigators. This result is consistent with the effects of MB on health observed in previous studies. Negative MB effects on the central nervous system and autonomic nervous system of asymptotic workers exposed to MB were recently reported (11, 12). In addition, the environment in the same workplace as that in the present study was exposed to almost 1,000 ppm MB in the proximities of fumigation containers during injection and degassing processes, which is much higher than the permissible exposure limit of 1 ppm for MB (43). It can be inferred that the elevated MB concentration has resulted in increased Br^−^ concentration in the urine of workers, thereby exerting a detrimental impact on their cardiovascular health.

The effects of MB on SDPTG indices and Br^−^ levels before and after work have been compared between the two groups while controlling for pre-work SDPTG and Br^−^ concentrations. The parameters d/a, e/a, and Br^−^ concentrations showed significant trend differences between the two groups. Considering that b/a and c/a, which reflect the early systolic component, did not show any differences between the two groups, it can be presumed that MB affects peripheral artery compliance more than aortic distensibility.

Despite all fumigators reporting the use of gas masks, as indicated in Table 1, their health still was adversely effected. Several factors may have contributed to MB exposure. Korean phytosanitary treatment regulations mandate that fumigators wear gas masks during fumigation tasks (33). While quarantine inspectors supervise the fumigation process on-site, their oversight is primarily focused on the moments when MB is applied or just before the gas is released. Fumigators engaged in other tasks may not wear masks due to inconveniences like impaired visibility or breathing difficulties. An earlier study also identified another sources of MB exposure (16). The gas mask can develop leaks at contact points with the face. Additionally, the use of inadequate respirator canisters during fumigation work heightens the risk of exposure. Two kinds of canisters can be affixed to the gas mask: MB and organic vapor (OV). The OV canister has a shorter time before breakthrough compared to the MB canister, and any absorbed MB within the OV canister can be emitted during breathing. In Korea, all fumigators employ OV canisters, which may elevate the likelihood of fumigation workers encountering greater MB concentrations.

Due to strict workplace access control, the selection of participants and locations for this study was challenging. Nonetheless, this study enhances our understanding of the health consequences of MB exposure on fumigators, who do not display any symptoms. However, this study has also some limitations, one of which is the relatively small sample size of 44 fumigators and 20 inspectors because only 76 fumigators and 36 inspectors were registered in the Busan Port area with the APQA. Additionally, our data do not explain the disparity of the c/a ratio of fumigators compared to the aligned trends of other indices. Furthermore, we remain uncertain whether the alteration in SDPTG observed in workers is a result of secondary changes stemming from the neurotoxicity of MB or its direct cardiovascular toxicity. Future studies should thoroughly examine the impact of MB on the arterial system, as well as its long-term effects on the health of fumigators. Despite these limitations, this study found evidence of the negative effects on the health of workers exposed to MB, including elevated urinary Br^−^ levels and altered SDPTG indices after work. The findings of this study have practical implications for enhancing fumigation procedures, mitigating hazards, and safeguarding the well-being of the employees. Additionally, they establish the groundwork for restricting the usage of MB and advocating the use of alternative approaches.

## 5. Conclusion

We evaluated 44 fumigators (study group) and 20 inspectors (control group) by measuring their SDPTG indices and urinary Br*^−^* concentrations before and after fumigation. These indices serve as indicators of vascular load and aging. The post-work SDPTG indices (b/a, c/a, d/a, e/a, and SDPTG-AI) and Br^−^ levels of the fumigators demonstrated significant changes compared to their pre-work values, indicating a negative impact of MB on cardiovascular health. However, the SDPTG indices and Br^−^ levels of the inspectors did not exhibit such differences. We also observed in all individuals’ significant correlations between Br^−^ levels and all SDPTG indices except c/a. These findings suggest that in the fumigator group, Br^−^ levels and SDPTG indices varied after work involving MB, causing detrimental effects on their health despite the absence of symptoms.

## Data availability statement

The data analyzed in this study is subject to the following licenses/restrictions: All the data supporting the findings of this study are available from the corresponding authors upon reasonable request. Requests to access these datasets should be directed to pmg@korea.kr.

## Ethics statement

The studies involving humans were approved by Institutional Review Board of Dong-A University (IRB number: 2-1040709-AB-N-01-201806-BR-004-04). The studies were conducted in accordance with the local legislation and institutional requirements. The participants provided their written informed consent to participate in this study.

## Author contributions

JC conceived and designed the study. M-GP wrote the first draft of the manuscript. JC and M-GP reviewed the manuscript. WC contributed to data acquisition. All authors contributed to the article and approved the submitted version.

## Funding

This study was supported by a National Research Foundation of Korea (NRF) grant funded by the Korean government (MSIT) (No. NRF-2022R1F1A1074196). This research was also supported by “Research Base Construction Fund Support Program” funded by Jeonbuk National University in 2023.

## Conflict of interest

The authors declare that the research was conducted in the absence of any commercial or financial relationships that could be construed as a potential conflict of interest.

## Publisher’s note

All claims expressed in this article are solely those of the authors and do not necessarily represent those of their affiliated organizations, or those of the publisher, the editors and the reviewers. Any product that may be evaluated in this article, or claim that may be made by its manufacturer, is not guaranteed or endorsed by the publisher.
