# Supplementary material for: Evaluation of the effect of photoplethysmograms on workers’ exposure to methyl bromide using second derivative
Source: Front Public Health. 2023 Sep 25;11:1224143. doi: 10.3389/fpubh.2023.1224143 (PMC10560719; doi:10.3389/fpubh.2023.1224143)
Supplement: Supplementary file 1 [file Table_1.docx]

Supplementary Material

Second derivative of photoplethysmogram analysis on workers exposed to methyl bromide

**1) Table S1. General work and SDPTG measurement processes of the subjects**

| Time | Fumigator | Inspector |
| --- | --- | --- |
| 08:00 | **Measure SDPTG indices**  **after stabilization** | - |
| 08:30 | - | **Measure SDPTG indices**  **after stabilization** |
| 09:00 | Move to working area | Prepare documents and travel to work area |
| 09:30 | Prepare MB injections  - Calculate MB dose  - Connect hose  - Seal container | Inspect plants  - Check documents and plants  - Open packing materials  - Visually inspect plants by cutting or sifting |
| 11:00 | Inject MB ^1^ | Oversee MB injections |
| 11:30 | Completion of MB injections | Complete overseeing MB injections |
| 12:00 | Lunch | Lunch |
| 13:30 | Degas MB  - Measure MB concentration  - Remove tape on containers  - Open containers  - Wait for MB concentration reduction (approximately 2hrs) | Confirm MB concentrations(~13:40)  Inspect plants (13:40~)  - Check document and plants  - Open packing materials  - Visually inspect plants by cutting or sifting |
| 15:30 | Travel back to office | Travel back to office |
| 16:00 | **Measure SDPTG indices**  **after stabilization** | Document findings |
| 16:30 | - | **Measure SDPTG indices**  **after stabilization** |

Subjects’ urine was collected just before the measurement of SDPTG indices. SDPTG indices and urinary Br- concentrations were measured in one or two subjects per group on the day of MB fumigation work.

^1^ MB was injected with 33-73 g/m3 (1).

1. Park M-G, Hong Y-S, Park CG, Gu D-C, Mo H-h. Variations in Methyl Bromide Concentration with Distance and Time During Quarantine Fumigation. *Environmental monitoring and assessment* (2021) 193(7):1-7.
